# Pollen/TLR4 Innate Immunity Signaling Initiates IL-33/ST2/Th2 Pathways in Allergic Inflammation

**DOI:** 10.1038/srep36150

**Published:** 2016-10-31

**Authors:** Jin Li, Lili Zhang, Xin Chen, Ding Chen, Xia Hua, Fang Bian, Ruzhi Deng, Fan Lu, Zhijie Li, Stephen C. Pflugfelder, De-Quan Li

**Affiliations:** 1 School of Optometry and Ophthalmology, Wenzhou Medical University, Wenzhou, China; 2 Ocular Surface Center, Cullen Eye Institute, Department of Ophthalmology, Baylor College of Medicine, Houston, Texas, USA; 3 Department of Pediatrics, Baylor College of Medicine, Houston, Texas, USA

## Abstract

Innate immunity has been extended to respond environmental pathogen other than microbial components. Here we explore a novel pollen/TLR4 innate immunity in allergic inflammation. In experimental allergic conjunctivitis induced by short ragweed (SRW) pollen, typical allergic signs, stimulated IL-33/ST2 signaling and overproduced Th2 cytokine were observed in ocular surface, cervical lymph nodes and isolated CD4^+^ T cells of BALB/c mice. These clinical, cellular and molecular changes were significantly reduced/eliminated in TLR4 deficient (*Tlr4-d*) or MyD88 knockout (*MyD88*^*−*/*−*^) mice. Aqueous SRW extract (SRWe) directly stimulated IL-33 mRNA and protein expression by corneal epithelium and conjunctiva in wild type, but not in *Tlr4-d* or *MyD88*^*−*/*−*^ mice with topical challenge. Furthermore, SRWe-stimulated IL-33 production was blocked by TLR4 antibody and NF-kB inhibitor in mouse and human corneal epithelial cells. These findings for the first time uncovered a novel mechanism by which SRW pollen initiates TLR4-dependent IL-33/ST2 signaling that triggers Th2-dominant allergic inflammation.

Increasing evidence suggests that the concept of innate immunity has been largely changed. Traditionally, the essential role of innate immunity is recognition of invading microorganisms, a process mediated by germ line-encoded pattern recognition receptors (PRRs), such as Toll-like receptors (TLRs), NOD-like receptors (NLRs), etc.^1^,^2^. Recent breakthroughs reveal that the innate immunity has been expanded to recognize and respond to environmental pathogen associated molecular patterns (PAMPs) other than conserved microbial components.

Furthermore, the advances in innate immunity have also changed the view on epithelial cells. In addition to a long-recognized property of their physical barrier function, epithelial cells are known to play important roles in initiation and regulation of immune responses. Epithelial cells are now recognized to participate in innate and adaptive immune responses, as well as in the transition from innate immunity to adaptive immunity (see review article^3^). For example, allergic disease is increasingly being seen as an epithelial disease both structurally and functionally, based on recent advances that epithelial cells play a vital role in innate immunity and serve as a bridge linking innate to adaptive immune responses.

Traditionally, much of research efforts have focused on the cells and molecules that mediate adaptive immunity, and have identified Th2 cell dominant disorder in most allergic inflammatory diseases. However, the underlying mechanism for initiation of this adaptive immune disorder by mucosal epithelium remains a relative mystery. A recently major breakthrough has been explored that epithelium-derived pro-allergic cytokines are key initiators in allergic inflammatory diseases. Interleukin (IL) 33 is just such a novel pro-allergic epithelial cytokine. IL-33, a newly discovered IL-1 family cytokine, was recently identified as a functional ligand to IL-1 receptor like 1 (IL1RL1), also referred as to ST2, which has been well known as a receptor on Th2 cells to mediate allergic inflammatory diseases^4^,^5^,^6^,^7^. IL-33 has been now recognized to trigger asthma, rhinitis, atopic dermatitis and allergic conjunctivitis^6^,^8^,^9^,^10^. Recently, the role of IL-33 in initiating allergic inflammation has been investigated in SRW pollen-induced mouse models of allergic conjunctivitis^11^,^12^ or allergic rhinitis^13^. The studies of Asada and Haenuki were carried out using IL-33 knockout mice and showed the pathophysiological roles of IL-33 in SRW induced allergic disease models in mouse^12^,^13^. However, it remains unknown whether and how IL-33 is induced by pollen allergens via TLR4-dependent innate immunity pathways.

Pollen is a ubiquitous allergen that affects a large population with allergic diseases. However, the mechanisms leading to resolution of pollen allergen-induced inflammation remain poorly understood. This represents an important challenge for us to resolve allergen-driven inflammation, which potentially leads to recurrent or chronic allergic diseases. Ambrosia artemisiifolia short ragweed (SRW) is the most widespread plant in North America. SRW pollen induced allergic conjunctivitis is a good model to study allergic diseases.

The present study uncovers a novel pollen/TLR4 innate immunity pathway where SRW pollen triggers allergic inflammation via TLR4-dependent innate immunity by mucosal epithelium, which produces pro-allergic cytokine IL-33 that activates IL-33/ST2/Th2 signaling pathways. Three *in vivo* and *in vitro* models were used in this study, a well-characterized murine model of experimental allergic conjunctivitis (EAC) induced by SRW pollen in BALB/c, TLR4 deficient (*Tlr4-d*) and MyD88 knockout (*MyD88*^*−*/*−*^) mice, a topical ocular surface challenge murine model, and a culture model of primary human corneal epithelial cells (HCECs) exposed to an aqueous extract of defatted SRW pollen (SRWe).

## Results

### IL-33/ST2 signaling triggers Th2 dominant inflammation in BALB/c mice with acute EAC induced by SRW pollen

To explore the role of epithelial pro-allergic cytokine IL-33 and its signaling pathways in pollen-triggered allergic inflammation, acute EAC model was induced in BALB/c mice sensitized and topically challenged by SRW pollen (SRW-EAC mice), with PBS-treated (PBS mice) and untreated mice as control groups. Topical challenge once with SRW allergen generated acute clinical signs mimic to human allergic conjunctivitis, including lid edema, conjunctival redness, chemosis, tearing, and frequent scratching of the eye lids. These signs were detected within 20 minutes, reached peak levels around 30–40 minutes, and persisted for 4–6 hours after SRW challenge. The scratching behavior and clinical signs lasted overnight or longer in some cases.

**Figure 1:**
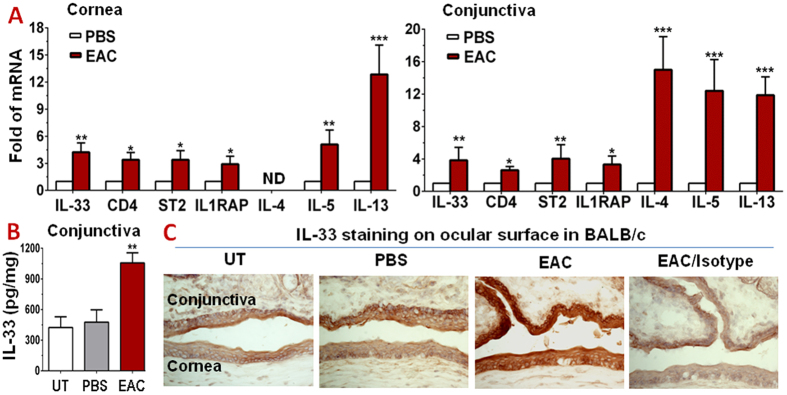
IL-33/ST2 signaling pathways are induced in acute EAC mice by SRW pollen compared with PBS control mice. (**A**) The stimulated mRNA expression of IL-33/ST2 signaling molecules and Th2 cytokines by corneal epithelium and conjunctiva in SRW-EAC BALB/c mice, evaluated by RT-qPCR. (**B**) Increased IL-33 protein levels in conjunctiva of EAC mice by ELISA. (**C**) Increased IL-33 production in ocular epithelium of EAC mice by immunohistochemical staining. Results shown are the Mean ± SD of four independent experiments. *P < 0.05, **P < 0.01, ***P < *0.001*, n = 4. ND: not detected.

Compared with the PBS control mice, IL-33 transcripts were significantly upregulated in the corneal epithelium (4.24 ± 1.03 fold) and conjunctiva (3.84 ± 1.38 fold) (all *P* < *0.01*) of SRW-EAC mice, as evaluated by RT-qPCR (Fig. 1A). Conjunctival IL-33 protein levels increased to 1056.34 ± 98.83 pg/mg cellular proteins (*P* < *0.01*) compared with the untreated (424.22 ± 104.79 pg/mg) and PBS (476.57 ± 121.01 pg/mg) control mice, as evaluated by enzyme-linked immunosorbent assay (ELISA) (Fig. 1B). Immunohistochemical staining further confirmed the induced production and localization of IL-33 protein in the SRW-challenged eyes of EAC model. IL-33 immunoreactivity was weakly localized in some basal and suprabasal cells of ocular surface epithelium in the untreated and PBS mice. The corneal and conjunctival epithelia in EAC mice displayed much stronger IL-33 staining throughout entire epithelium when compared with isotype IgG negative control (Fig. 1C). These data indicate that IL-33 expression was largely induced at both mRNA and protein levels in ocular surface epithelia in this SRW-induced EAC model.

**Figure 2:**
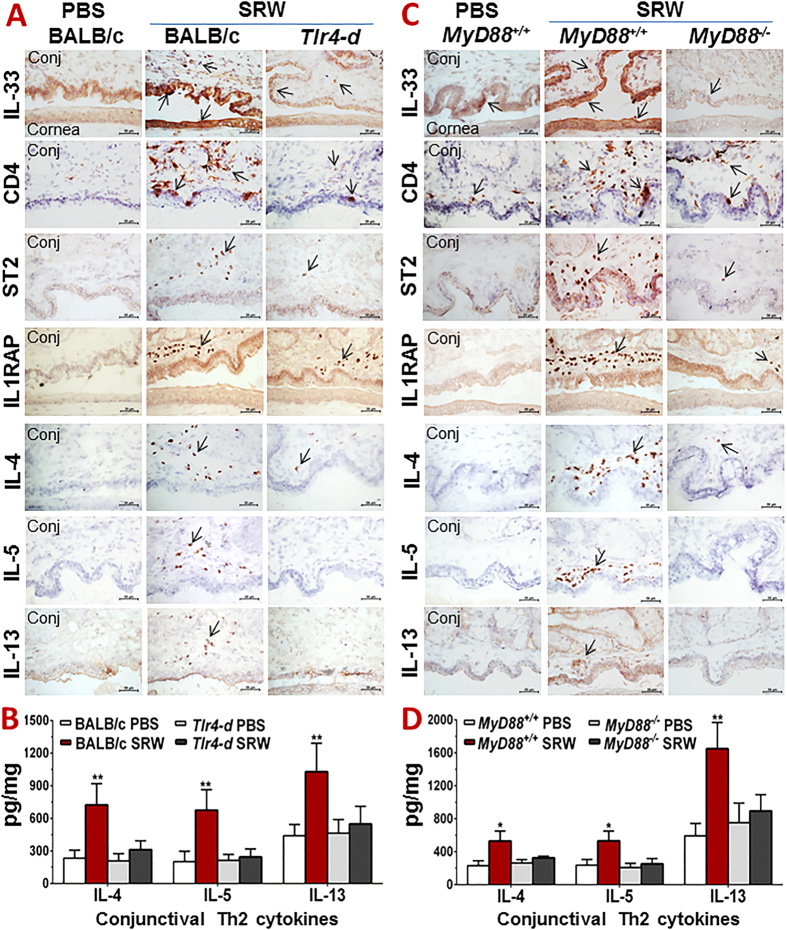
The stimulated production of IL-33 in SRW-induced EAC model requires TLR4 and MyD88. (**A,C**) Immunohistochemical staining of IL-33 and its signaling molecules and Th2 cytokines on cornea and conjunctiva (Conj) in wild type BALB/c and *Tlr4-d* mice (**A**), as well as in *MyD88*^+/+^ and *MyD88*^*−*/*−*^ mice (**C**), challenged by SRW pollen, with PBS-treated mice as controls. Bar: 20 μm; Arrows: positive staining signals. (**B,D**) Protein levels of Th2 cytokines by ELISA in conjunctiva of wild type BALB/c and *Tlr4-d* mice (**B**), as well as in *MyD88*^+/+^ and *MyD88*^*−*/*−*^ mice (**D**). Results shown are Mean ± SD. *P < 0.05, **P < *0.01*; n = 4, compared with PBS controls.

The accumulation of CD4^+^ T cells in ocular surface was observed in this EAC model. As shown in Fig. 1A, the levels of transcripts coding for CD4, ST2 (IL1RL1) and IL-1 receptor accessory protein (IL1RAP) increased significantly in the ocular surface. Their mRNA expression increased to 2.62 ± 0.45, 4.03 ± 1.73 or 3.32 ± 1.03 fold, respectively (all *P* < *0.05*) in conjunctiva of SRW-challenged EAC mice, compared with PBS mice. In addition, the transcripts coding for Th2 cytokines, IL-4, IL-5 and IL-13, were dramatically upregulated to 15.0 ± 4.08, 12.4 ± 3.85, and 11.9 ± 2.27 fold (all *P* < *0.001*), respectively, in conjunctiva of EAC mice (Fig. 1A). IL-5 and IL-13 transcripts also increased in corneal epithelium maybe due to Th2 cell infiltration. These results suggest that the infiltrated CD4^+^ T cells in conjunctiva appear to be Th2 lineage, a result from stimulation of IL-33, which activated T cell receptors ST2 and IL1RAP, and promoted differentiation of naïve T cells to Th2 cells that express IL-4, IL-5 and IL-13. The immunohistochemical staining further confirmed the dramatically induced IL-33 production by ocular surface epithelia, largely infiltrated CD4^+^ T cells with activated ST2 and IL1RAP, as well as Th2 cytokines IL-4, IL-5 and IL-13 in conjunctival stroma of SRW-induced EAC mice when compared with PBS mice (Fig. 2A). The protein levels of IL-4, IL-5 and IL-13 increased to 722.69 ± 196.07, 674.81 ± 189.17, and 1028.10 ± 261.96 pg/mg cellular proteins (All *P* < *0.01*), respectively, in conjunctival tissue lysates of SRW mice, compared with 233.67 ± 73.59, 201.94 ± 95.10, and 439.84 ± 103.68 pg/mg in PBS mice, as evaluated by ELISA (Fig. 2B). All these findings suggest that IL-33/ST2 signaling triggers Th2-dominant inflammatory response on ocular surface in SRW-challenged EAC mice.

### Epithelial IL-33 triggers Th2 inflammatory response through T cell receptors ST2 and IL1RAP

**Figure 3:**
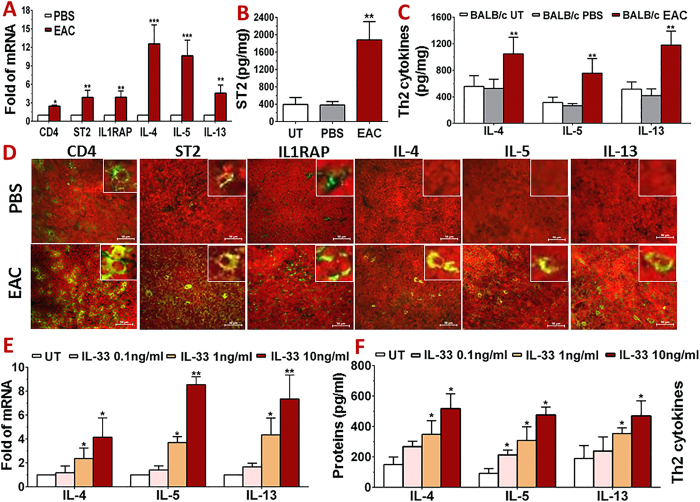
IL-33 triggers Th2 inflammatory response in CLNs of SRW-EAC BALB/c mice with PBS mice as controls. (**A**) The upregulated mRNA of Th2 cell surface markers and cytokines by CLNs, evaluated by RT-qPCR. (**B,C**) Protein levels of ST2 and Th2 cytokines in CLNs by ELISA. (**D**) Immunofluorescent staining of Th2 cell markers and cytokines in CLNs. (**E,F**) Direct effects of IL-33 on Th2 cytokine expression by isolated CD4^+^ T cells at mRNA (**E**) and protein levels (**F**). Results shown are Mean ± SD. *P < 0.05, **P < 0.01 ***P < *0.001*; n = 4, compared with PBS or untreated (UT) controls.

To confirm that epithelium-derived IL-33 bridges adaptive immune response to trigger allergic inflammation by promoting differentiation of naïve T cells to Th2 lineage in SRW-induced EAC mice, the ocular surface draining cervical lymph nodes (CLNs) were collected for evaluation. Compared with PBS mice, CD4 expression increased to 2.45 ± 0.18 fold, accompanied by significantly upregulated transcripts of ST2 (3.87 ± 1.35 fold) and IL-1RAP (3.90 ± 1.02 fold) in CLNs of EAC mice. The mRNA expression of Th2 cytokines, IL-4, IL-5 and IL-13, markedly increased to 12.56 ± 3.06 (*P* < *0.001*), 10.6 ± 2.54 (*P* < *0.001*) and 4.6 ± 1.34 fold (*P* < *0.01*), respectively (Fig. 3A). ELISA data confirmed the large upregulation (all *P* < *0.01*) of ST2 (1882.55 ± 422.64 pg/mg), IL-4 (1047.07 ± 250.12 pg/mg), IL-5 (755.51 ± 220.54 pg/mg) and IL-13 (1179.70 ± 210.31 pg/mg) at protein levels in CLN lysates of EAC mice when compared with untreated and PBS control mice (Fig. 3B,C).

The increased number of CD4^+^, ST2^+^ or IL1RAP^+^ cells in the draining CLNs of EAC mice was further confirmed by immunofluorescent staining. As shown in Fig. 3D, the immunoreactivity and positive cell number of these 3 markers for Th2 cells dramatically increased, which were accompanied by increased staining of Th2 cytokines in CLNs of EAC mice, when compared to PBS control mice. All these results suggest that naïve CD4^+^ T cells in CLN were largely differentiated to Th2 cells in response to ocular surface epithelium-derived IL-33 in acute EAC induced by SRW pollen.

To investigate the direct effects of IL-33 on T cells, CD4^+^ cells were isolated from CLNs and spleens of SRW-EAC mice, and treated with different concentrations of recombinant mouse IL-33. As shown in Fig. 3E, the mRNA expression of Th2 cytokines IL-4, IL-5 and IL-13 was dose-dependently upregulated by IL-33 at 0.1, 1 and 10 ng/ml. ELISA data confirmed these stimulatory effects of IL-33 on Th2 cytokines at protein levels (Fig. 3F). The protein production of IL-4, IL-5 and IL-13 from untreated control (150.15 ± 49.94, 92.41 ± 31.10, and 190.21 ± 84.21 pg/ml) significantly increased to 348.43~517.86, 308.39~476.23 and 354.17~469.42 pg/ml, respectively, in the culture media of T cells treated by 1 and 10 ng/ml of IL-33.

### IL-33/ST2-triggered allergic inflammation was initiated by Pollen/TLR4 innate immunity signaling in SRW-EAC model

IL-33 induction has been recognized to be involved in TLR-mediated innate immunity^14^. Our previous studies also showed that IL-33 was largely induced by human corneal epithelial cells in response to certain TLR ligands^15^. MyD88 is a universal adapter protein essential for responding to most TLRs except TLR3^16^,^17^. *Tlr4*-deficient (*Tlr4-d*, C.C3-Tlr4^Lps-d^/J) and *MyD88*^*−*/*−*^ mice are useful to determine whether TLR4 signaling is involved in innate response^18^,^19^. To explore whether SRW pollen initiates IL-33 through TLR4-dependent innate response, we sensitized and topically challenged the *Tlr4-d* and *MyD88*^*−*/*−*^ mice with SRW pollen using the protocol same to making EAC model in BABL/c mice.

**Figure 4:**
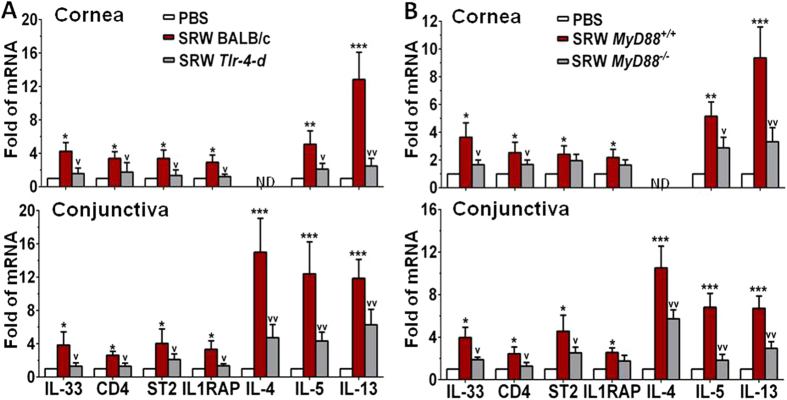
IL-33 induction in SRW-induced EAC model requires TLR4 and MyD88. The mRNA expression of IL-33 and its signaling molecules and Th2 cytokines by corneal epithelium and conjunctiva in EAC model with BALB/c and *Tlr4-d* mice (**A**), as well as in C57BL/6 based *MyD88*^+/+^ and *MyD88*^*−*/*−*^ mice (**B**), with PBS-treated mice as controls. Results shown are the Mean ± SD. *P < 0.05, **P < 0.01, ***P < *0.001*; n = 4, compared with PBS controls; ^*v*^*P* < *0.05,*^*vv*^*P* < *0.01,* n = 4, compared with wild type mice.

Compared with wild-type BALB/c mice, the ocular allergic signs, the stimulated IL-33 and its receptors ST2 and IL1RAP, as well as the largely induced Th2 cytokines in ocular mucosa, especially conjunctiva, were dramatically reduced or eliminated in *Tlr4-d* mice. The clinical signs of lid edema, conjunctival redness and chemosis, the stimulated production of IL-33, CD4, ST2, IL1RAP and Th2 cytokines IL-4, IL-5 and IL-13, shown by immunohistochemical staining (Fig. 2A) and ELISA (Fig. 2B), and their significantly induced mRNA levels in corneal epithelium and conjunctiva (Fig. 4A) were only observed in wild-type BALB/c, but not in *Tlr4-d* mice. These findings suggest that SRW pollen initiates IL-33 induction and signaling for allergic inflammation was through TLR4-dependent innate immune response in EAC mice.

The typical allergic signs and IL-33/ST2/Th2 signaling molecular changes were also observed in C57BL/6 based wild type *MyD88*^+/+^ mice. The clinical signs, the immunoreactivity (Fig. 2C) and protein levels (Fig. 2D) of IL-33, ST2, IL1RAP and Th2 cytokines IL-4, IL-5 and IL-13 as well as their mRNA levels (Fig. 4B) were significantly stimulated in ocular surface of SRW-challenged *MyD88*^+/+^ mice, but dramatically reduced or eliminated in SRW challenged *MyD88*^*−*/*−*^ mice. These findings suggest that MyD88 pathway is involved in the TLR4-dependent IL-33 signaling induced by SRW pollen.

### IL-33 induction by SRW in ocular surface was TLR4-dependent innate immune response in a topical challenge murine model

**Figure 5:**
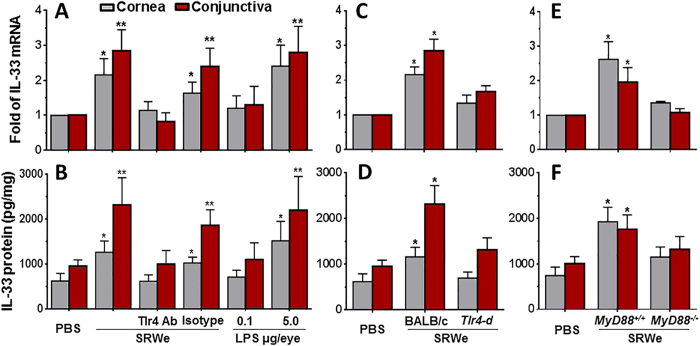
IL-33 induction by SRW in ocular surface is TLR4-dependent in a topical challenge murine model. (**A,B**) BALB/c mice were topically instilled with SRWe at 150 μg/5 μl/eye without or with pre-instilled rat anti-mouse TLR4 antibody (1 μg/5 μl/eye) or its isotype rat IgG2a, and LPS at 5 μg/5 μl/eye or 0.1 μg/5 μl/eye was used as positive control. (**C,D**) IL-33 induction by topically challenged SRWe in wild type BALB/C and *Tlr4-d* mice. (**E,F**) IL-33 induction by topically challenge in *MyD88*^+/+^ and *MyD88*^*−*/*−*^ mice. (**A,C,E**) Show IL-33 mRNA expression by RT-qPCR, and (**B,D,F**) show its protein levels by ELISA. Results shown are Mean ± SD (n = 5). *P < 0.05, **P < *0.01,* compared with PBS controls.

To confirm that SRW pollen directly induces IL-33 expression by ocular mucosal epithelia through TLR4-dependent innate immunity pathway, we created a topical challenge murine model using SRWe at 150 μg/5 μl/eye for 4–24 hours^20^. SRWe induced IL-33 mRNA at 8 hours, and increased IL-33 protein levels in 24 hours by ocular epithelia exposed to SRWe. As shown in Fig. 5A, IL-33 mRNA was significantly upregulated to 2.16 ± 0.46 and 2.85 ± 0.60 fold (both *P* < *0.05*), respectively, in corneal epithelium and conjunctiva by SRWe in BALB/c mice, as compared with PBS-treated controls. Evaluated by ELISA (Fig. 5B), IL-33 protein level increased by 2.46 fold (from 956.52 ± 133.83 to 2318.52 ± 603.47 pg/mg cellular protein) in conjunctiva of BALB/c mice exposed to SRWe. The stimulatory levels of IL-33 mRNA and protein by SRW were similar to the levels by LPS at 5 μg/5 μl/eye while LPS at 0.1 μg/5 μl/eye did not show significant effect (Fig. 5A,B). Furthermore, SRWe-induced IL-33 was largely blocked at both mRNA and protein levels by a TLR4 antibody but not by its isotype rat IgG2a.

Interestingly, IL-33 mRNA (Fig. 5C) and protein levels (Fig. 5D) did not increase in corneal epithelium and conjunctiva of *Tlr4-d* mice by SRWe topical challenge. Furthermore, we applied this topical challenge model to *MyD88*^*−*/*−*^ mice and their wild type controls. Interestingly, SRWe promoted IL-33 expression by ocular surface at both mRNA (Fig. 5E) and protein levels (Fig. 5F) only in *MyD88*^+/+^ mice, but not in *MyD88*^−/−^ mice. Taken together, these findings demonstrate that SRWe directly stimulates IL-33 expression by ocular mucosal epithelia via a TLR4-dependent innate pathway.

### IL-33 induction by SRW was TLR4-dependent innate immune response in HCEC culture model

**Figure 6:**
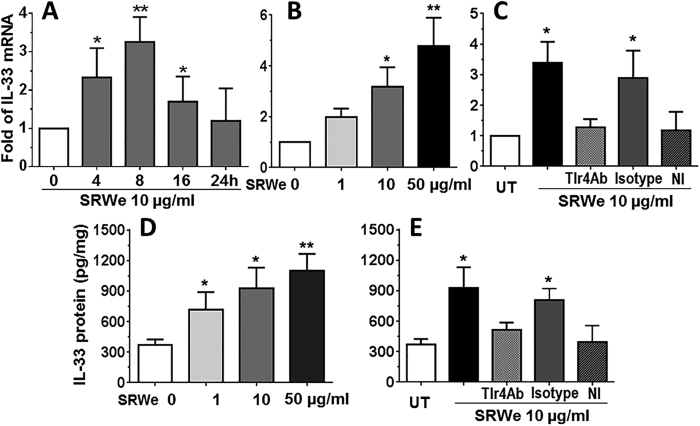
IL-33 induction by SRW was TLR4-dependent in HCEC culture model. (**A–C**) Primary HCECs were treated with different concentrations of SRWe (1–50 μg/ml) for 0–24 hours to detect IL-33 mRNA and protein production. (**D,E**) HCECs were pre-incubated with mouse TLR4 antibody (5 μg/ml), isotype mouse IgG2a k, or NF-kB Activation Inhibitor quinazoline (NI, 10 μM) for 1 hour before adding 10 μg/ml SRWe for IL-33 expression at mRNA and protein levels. Results shown are Mean ± SD (n = 5). *P < 0.05, **P < *0.01*, compared with untreated cells.

To identify this phenomenon in humans, we investigated pollen/TLR4 signaling in IL-33 expression by primary cultured HCECs. The time course experiments showed that the IL-33 mRNA expression was stimulated to peak levels at 8 hours in HCECs by SRWe at 10 μg/ml (Fig. 6A). We observed the concentration-dependent upregulation of IL-33 mRNA at 8 hours (Fig. 6B) and its protein level at 48 hours (Fig. 6C) in primary HCECs exposed to SRWe (1–50 μg/ml). IL-33 protein production increased to 719.10 ± 170.64 (*P* < *0.05*), 926.33 ± 204.22 (*P* < *0.05*) and 1100.30 ± 164.84 (*P* < *0.01*) pg/mg cellular proteins respectively in HCECs exposed to SRWe at 1, 10, 50 μg/ml, compared with 370.78 ± 54.13 pg/mg in untreated controls. Interestingly, the SRWe (10 μg/ml)-stimulated IL-33 mRNA (Fig. 6D) and protein (Fig. 6E) levels were largely blocked by pre-incubation of cells with 5 μg/ml of neutralizing monoclonal antibody against human TLR4, but not by its isotype mouse IgG2a k. SRWe stimulated IL-33 expression was also significantly inhibited by quinazoline, a NF-kB activation inhibitor (NI). These findings suggest that SRW induces IL-33 production in HCECs through TLR4-dependent innate signaling pathway.

## Discussion

Pollen is a ubiquitous allergen that affects a large population. However, the molecular mechanism by which pollen allergens initiate innate immune responses to trigger allergic inflammation is largely unknown, a knowledge gap preventing us from developing new targeted therapies to cure allergic diseases. TLRs are major pattern recognition receptors that initiate innate immune responses to pathogen- or damage-associated molecular patterns (PAMPs and DAMPs). For examples, TLR4 is such a novel one. TLR4 recognizes more various ligands than previous thought. In addition to first identified bacterial LPS, TLR4 was known to recognize non-microbial sources, such as protein extracts from plants and herbs. Taxol, an antitumor agent derived from the Yew plant, was shown to activate mouse macrophages through TLR4^21^, and aqueous extract of Rhodiola imbricata rhizome, a medicinal plant, was reported to stimulate TLR4 innate response^22^.

Recently, Chung and colleagues revealed that LPS suppresses Th2 responses via the TLR4-dependent pathway in the ovalbumin induced EAC model^23^. LPS administration markedly suppressed IgE-mediated and eosinophil-dependent conjunctival inflammation. Mice sensitized with ovalbumin plus LPS had less IL-4, IL-5 and eotaxin secretion than mice sensitized with ovalbumin only, suggesting that LPS/TLR4 signaling shifts Th2 to Th1 response. Interestingly, the present study uncovers a novel mechanism by which SRW pollen initiates TLR4-dependent IL-33/ST2 signaling that triggers Th2-dominant allergic inflammation, suggesting that pollen/TLR4 signaling promotes Th2 response.

Firstly we demonstrated the IL-33/ST2 signaling pathway that triggers Th2-dominant allergic inflammation in a well-characterized murine EAC model by SRW pollen^24^,^25^,^26^. SRW pollen is a common environmental allergen that causes a variety of allergic diseases including asthma, rhinitis, dermatitis and conjunctivitis in large populations of North American people. Thus SRW has been widely used for allergic models including asthma, rhinitis and skin allergy, in mouse, guinea-pig, dog and other animal species^27^,^28^,^29^. However, there is no report showing how SRW pollen triggers IL-33/ST2 signaling although ST2 has been well known as a receptor on Th2 cells to mediate allergic inflammation^4^,^5^,^6^,^7^. Our results showed that IL-33 expression was largely induced at both mRNA and protein levels in corneal and conjunctival epithelia (Figs 1,2 and 4), the infiltrated CD4^+^ T cells in conjunctiva were largely in Th2 lineage expressing IL-4, IL-5 and IL-13 with activated Th2 cell receptors ST2 and IL1RAP (Fig. 2), and CD4^+^ T cells in the ocular surface draining CLNs were also differentiated to Th2 cells in response to ocular surface epithelium-derived IL-33 (Fig. 3) in acute EAC model induced by SRW pollen. To explore the direct effects of IL-33 on T cells, we further showed that mrIL-33 dose-dependently stimulated expression at both mRNA and protein levels of Th2 cytokines IL-4, IL-5, and IL-13 (Fig. 3E,F).

Secondly, we demonstrated that IL-33/ST2 allergic pathway is initiated by pollen/TLR4 innate immunity signaling in Th2-dominant inflammation. This novel mechanism was revealed by strong evidence that all clinical signs of allergic conjunctivitis, stimulated expression of IL-33 by ocular epithelium, upregulated Th2 cell receptors ST2 and IL1RAP, as well as Th2 cytokine-dominant inflammation were only observed in EAC model of wild type BALB/c and *MyD88*^+/+^ mice, but not in *Tlr4-d* or *MyD88*^*−*/*−*^ mice with the same treatment by SRW sensitization and ocular surface challenge (Figs 2, 3, 4).

Thirdly, we demonstrated the direct evidence that SRW stimulates IL-33 expression and production by ocular surface epithelium via TLR4-dependent innate immunity signaling using an *in vivo* mouse model with SRWe topical challenge. Our results showed that the aqueous protein extract of defatted SRW pollen significantly increased IL-33 expression at mRNA and protein levels in a concentration-dependent manner, and this stimulation was largely suppressed by exogenous TLR4 antibody in wild BALB/c mice. The direct stimulation of IL-33 expression by SRWe in corneal and conjunctival epithelia was only observed in the topical challenge model of wild type BALB/c and *MyD88*^+/+^ mice, but not in *Tlr4-d* or *MyD88*^*−*/*−*^ mice (Fig. 4).

Finally, we demonstrated that the pollen/TLR4 signaling is essential or indispensible for IL-33 induction by SRWe using an *in vitro* culture model of primary human corneal epithelial cells. The results showed that IL-33 expression at mRNA and protein levels was concentration-dependently upregulated by SRWe (1–50 μg/ml) in primary HCECs, and this stimulation was significantly blocked by a neutralizing antibody against human TLR4, but not by its isotype mouse IgG2a k (Fig. 6). Furthermore, SRWe-stimulated IL-33 expression was also significantly inhibited by quinazoline, a NF-kB Activation Inhibitor (Fig. 6). The findings support our previous reports^15^,^20^ and provide novel evidence to our hypothesis for humans that pollen/TLR4 signaling induces IL-33 expression via NF-kB pathway.

In conclusion, we have uncovered a novel mechanism by which pollen/TLR4 innate immunity signaling initiates IL-33/ST2 allergic pathway that triggers Th2-dominant inflammation in pollen induced allergic diseases. These findings provide new evidence that allergic conjunctivitis appears to be a mucosal epithelial disorder; and the innate immunity of epithelial cells not only recognize microbial invasion, but is also capable to respond to pollen components from plants. This discovery will shed light on the understanding of mucosal innate immunity involved in allergic inflammation, and may create new therapeutic targets to prevent and cure allergic disease.

## Material and Methods

### Animals

The animal research protocol was approved by the Institutional Animal Care and Use Committee (IACUC) at Baylor College of Medicine. All animals used in this study were maintained in specific pathogen-free conditions in microisolator cages and were treated in accordance with the guidelines provided in the Association for Research in Vision and Ophthalmology statement for the Use of Animals in Ophthalmic and Vision Research. BALB/c and BALB/c based homozygote *Tlr4* deficient (C.C3-*Tlr4*^Lps-d^/J) female mice aged at 6–8 weeks were purchased from the Jackson Laboratory (Bar Harbor, ME). The heterozygous MyD88 (myeloid differentiation primary response gene 88) knockout mice in C57BL/6 background were kindly provided by Dr. Shizuo Akira (Research Institute for Microbial Disease, Osaka University, Japan) through Dr. Eric Pearlman (Department of Ophthalmology and Visual Sciences, Case Western Reserve University, Cleveland, Ohio)^30^. The genotyping was performed by polymerase chain reaction (PCR) of the tail DNA with three specific primers (MyD88F: TGGCATGCCTCCATCATAGTTAACC; MyD88R: GTCAGAAACAACCACCACCATGC; MyD88R-Neo: ATCGCCTTCTATCGCCTTCTTGACG using a previous described method^20^. The age- and gender-matched *MyD88*^+/+^ littermates of the *MyD88*^*−*/*−*^ mice grown to 8–10 week old were used for experiments.

### Reagents

SRW pollen and the lyophilized aqueous extract of defatted short ragweed pollen were purchased from Greer Lab (Lenoir, NC). Amount of endotoxin in these SRW preparations were 0.00038 ± 0.00026 EU/μg protein, as measured by ToxinSensor^TM^ Chromogenic LAL Endotoxin Assay Kit (GenScript, Piscataway, NJ). Imject Alum was from Pierce Biotechnology (Rockford, IL). lipopolysaccharide (LPS) from Escherichia coli were from Sigma-Aldrich (St. Louis, MO). Recombinant mouse IL-33, human and mouse IL-33 ELISA kits were from BioLegend (San Diego, CA). Mouse ELISA kits for IL-4, IL-5 and IL-13 were from BosterBio (Pleasanton, CA). Rat anti-mouse CD4 primary antibody was from BD Pharmingen (San Jose, CA); rat anti-mouse IL-4 or IL-5 from Biolegend (San Diego, CA); rabbit anti-mouse IL-33 or ST2, and goat anti-mouse IL1RAP or IL-13 from Santa Cruz Biotechnology (Santa Cruz, CA) (see Supplemental Table 1 for details).

### Murine model of acute EAC induced by SRW pollen

The mouse EAC model was induced using previously reported methods^24^,^26^ with modification^31^. In brief, mice were immunized with 50 μg of SRW pollen (Greer Lab, Lenoir, NC) in 5 mg Imject Alum (Pierce Biotechnology, Rockford, IL) by footpad injection on day 0. Allergic conjunctivitis was induced by given topical applications of 1.5 mg SRW pollen suspended in 10 μl phosphate buffered saline (PBS), pH 7.2, into each eye once at day 11. Untreated normal and PBS eyedrop treated SRW-sensitized mice were used as controls. Animals were examined clinically for signs of immediate hypersensitivity responses 30 minutes after each topical challenge with SRW pollen. A clinical scoring scheme described by Magone and colleagues^24^ was used to evaluate chemosis, conjunctival redness, lid edema, and tearing. On day 12, 24 hours after the SRW challenge, the corneal epithelium, conjunctiva, cervical lymph nodes and whole eyes were harvested for gene expression assays and histopathological studies. Five mice per group were used in each experiment, and the same experiments were repeated at least 4 times.

### Murine model for IL-33 induction by SRWe topical challenge in ocular surface

As previous described^20^, mice were topically challenged with SRWe (Greer Lab) at 150 μg/5 μl/eye or 5 μl PBS as controls for different time periods (4–24 hours). SRWe was applied without or with pre-instilled rat anti-mouse TLR4 antibody (1 μg/5 μl/eye, eBioscience) or its isotype rat IgG2a. Tissue specimens including corneal epithelium and conjunctival tissues were collected at different time points and stored at −80 °C before used for IL-33 mRNA and protein assays. Four mice per group were used in each experiment, which was repeated 5 times.

### *In vitro* culture models for IL-33 induction by SRWe in primary (HCECs)

Fresh human corneoscleral tissues ( < 72 hours after death) from donors aged 21–56 years were obtained from the Lions Eye Bank of Texas (Houston, TX). HCECs were cultured in 12-well plates using explants from corneal limbal rims in a supplemented hormonal epidermal medium (SHEM) containing 5%FBS using our previous methods^32^. Confluent corneal epithelial cultures were switched to serum-free SHEM and treated for different time periods (4–48 hours) with different concentration of SRWe (1–50 μg/ml). SRWe at 10 μg/ml was applied without or with pre-incubated mouse anti-human TLR4 antibody (10 μg/ml, eBioscience), its isotype mouse IgG2a k, or quinazoline, an NF-kB Activation Inhibitor (NI, 10 μM, EMD Biosciences). Each experiment was repeated five times.

### *In vitro* culture models for direct effects of IL-33 on murine CD4^+^ cells

Murine CD4^+^ T cells were isolated from the spleens and cervical lymph nodes of 6–8 weeks old BALB/c mice as described previously^33^,^34^. In brief, the spleens and cervical lymph nodes of mice were surgically excised, crushed between two sterile frosted glass slides to make a single cell suspension while red blood cells were lysed with ammonium chloride. The cell suspension was centrifuged at 1000 rpm for 5 minutes, filtered and resuspended with rat anti-mouse CD4-conjugated magnetic microbeads diluted with cold 0.5% BSA in PBS (1:10 dilution, 90 μL of buffer and 10 μL of beads for every 1 × 10^7^ cells). After incubation for 15 minutes at 4 °C and washing, the cell were suspended in 500 uL buffer per 1 × 10^8^ cells, and loaded onto a micro-column. Positive cells attached to the column were removed with a plunger and the CD4^+^ T cells were cultured in RPMI 1640 supplemented with 10% fetal bovine serum (FBS). The CD4-enriched cell suspensions contained >80% CD4^+^ T cells as determined by flow cytometry.

The freshly isolated murine CD4^+^ T cells (1–5 × 10^6^/ml/well) were cultured in 24-well plates in RPMI 1640 with 10% FBS (Control) and treated for 4–48 hours with different concentrations (0.1–10 ng/ml) of rmIL-33. Medium supernatant was corrected and CD4^+^ T cells were lysed for total RNA extraction or cellular proteins, which were stored at −70 °C and used for RT-qPCR and ELISA of Th2 cytokines.

### Total RNA extraction, reverse transcription (RT) and quantitative real-time PCR (qPCR)

Total RNA was extracted with RNeasy Plus Mini Kit (Qiagen, Valencia, CA) according to the manufacturer’s instructions, quantified with a spectrophotometer (NanoDrop ND-2000; Thermo Scientific, Wilmington, DE), and stored at −80 °C before use. The first strand cDNA was synthesized by RT from 1.0 μg of total RNA using Ready-To-Go You-Prime First-Strand Beads as previously described^35^. Quantitative real-time PCR was performed in StepOnePlus™ Real-Time PCR System (Applied Biosystems, Foster City, CA) with 10 μl reaction volume containing 4 μl of cDNA, 0.3 μl TaqMan gene expression assay, 5 μl TaqMan gene expression master mix and 0.7 μl H_2_O. TaqMan gene expression assays used for this study were: human GAPDH (Hs99999905_m1) and IL-33 (Hs00369211_m1); and mouse GAPDH (Mm99999915_g1), IL-33 (Mm00505403_m1), ST2 (IL1RL1, Mm00516117_m1), IL-1R1Acp (Mm00492638_m1), CD11c (Mm00498698), CD4 (Mm00442754), IL-4 (Mm00442754), IL-5 (Mm0099999063), and IL-13 (Mm00434204). The thermocycler parameters were 50 °C for 2 min and 95 °C for 10 min, followed by 35 cycles of 95 °C for 15 s and 60 °C for 1 min. A non-template control was included to evaluate DNA contamination. The results were analyzed by the comparative threshold cycle (Ct) method and normalized by GAPDH as an internal control^35^,^36^.

### ELISA

Double-sandwich ELISA for human and mouse cytokines was performed to determine their protein levels in the cell lysates from mouse ocular specimens, CD4^+^ T cells and HCECs, as well as in culture supernatants, according to the manufacturer’s protocols similar to our previous report^15^,^37^. Absorbance was read at 450 nm with a reference wavelength of 570 nm by Infinite M200 microplate reader (Tecan US, Inc., Morrisville, NC).

### Immunohistochemical and immunofluorescent staining

The eyes and lids of mice in each group were excised, embedded in optimal cutting temperature (OCT) compound (VWR, Suwanee, GA), and flash-frozen in liquid nitrogen. Sagittal 8 μm cryosections from mouse globes were cut with a cryostat (HM 500; Micron, Waldorf, Germany), and stored at −80 °C before use. Immunohistochemical and immunofluorescent staining was performed as previously described^32^,^38^. Secondary antibody alone or isotype IgG were used as the negative controls. The results were photographed with an epifluorescence microscope (Eclipse 400; Nikon, Garden City, NY) using a digital camera (DMX 1200; Nikon).

### Statistical analysis

Student’s t-test was used to compare differences between two groups. One-way ANOVA test was used to make comparisons among three or more groups, followed by Dunnett’s post-hoc test. *P* values  < 0.05 were considered statistically significant.

## Additional Information

**How to cite this article**: Li, J. *et al*. Pollen/TLR4 Innate Immunity Signaling Initiates IL-33/ST2/Th2 Pathways in Allergic Inflammation. *Sci. Rep.* **6**, 36150; doi: 10.1038/srep36150 (2016).

**Publisher’s note:** Springer Nature remains neutral with regard to jurisdictional claims in published maps and institutional affiliations.

## Supplementary Material

Supplementary Information
