# Supplementary material for: Pollen/TLR4 Innate Immunity Signaling Initiates IL-33/ST2/Th2 Pathways in Allergic Inflammation
Source: Sci Rep. 2016 Oct 31;6:36150. doi: 10.1038/srep36150 (PMC5087075; doi:10.1038/srep36150)
Supplement: Supplementary Information [file srep36150-s1.pdf]

Pollen/TLR4 Innate Immunity Signaling Initiates IL-33/ST2/Th2 Pathways in Allergic Inflammation

Jin Li, M.D., Ph.D.,[1,2*] Lili Zhang, M.D., Ph.D.,[2*] Xin Chen, M.D.,[1,2] Ding Chen, M.D., O.D.,[1,2] Xia Hua, M.D., Ph.D.,[2] Fang Bian, M.D., Ph.D.,[2] Ruzhi Deng, M.D., Ph.D.,[1] Fan Lu, M.D., O.D.,[1**] Zhijie Li, M.D., Ph.D.,[3] Stephen C. Pflugfelder, M.D.,[2] De-Quan Li, M.D., Ph.D.[2**]

Table 1. Antibodies used for immunohistochemical staining

Antibodies	Company	Clones	Cat. No.	µg/ml	Dilution
CD4	BD Pharmingen	RM4-5	550280	62.5	1:50
IL-33	Santa Cruz	M-266	sc-98660	200	1:100
ST2	Santa Cruz	C-20	sc-18687	200	1:100
IL1RAP	Santa Cruz	N-20	sc-47056	200	1:100
IL-13	Santa Cruz	M-17	sc-1776	200	1:100
IL-4	Biolegend	11B11	504102	500	1:200
IL-5	Biolegend	TRFK5	504302	500	1:200
